# 

**Published:** 1896-09

**Authors:** 


					﻿Vol. XVI.
SEPTEMBER, 1896.
NO. 9.
THE
HOMEOPATHIC PHYSICIAN,
A Monthly Journal of Homœopathic Materia Medica
and Clinical Medicine.
“ He it a freeman whom truth makes free, and all are slaves beside."
"Seek the Truth: eome whence it may, cost what it will."
EDITED AND PUBLISHED BT
WALTER ML. JAMES, ME. D.
PHILADELPHIA :
TTo. 1231 LOOTST STREET.
I. o	x> O JST : •
ALFRED HEATH & CO., 8ole Agents fob! Gbeat Britain,
114 Ebury Street, Eaton Square.
-Yearly Subscription, $2.50, invariably in advance. Single numbers, 95 ets.
Entered'at the Philadelphia Po.t-Offlee aa 8econd.Cla»e Mail Matter.
				

